# Anti-Programmed Cell Death Protein 1 (PD-1) Immunotherapy for Metastatic Hepatocellular Carcinoma After Liver Transplantation: A Report of Three Cases

**DOI:** 10.7759/cureus.11150

**Published:** 2020-10-25

**Authors:** Ouissam Al Jarroudi, Ayhan Ulusakarya, Wathek Almohamad, Said Afqir, Jean-Francois Morere

**Affiliations:** 1 Medical Oncology, University Hospital Mohammed VI, Oujda, MAR; 2 Medical Oncology, Assistance Publique – Hôpitaux de Paris (AP-HP) Paul Brousse Hospital, Paris, FRA

**Keywords:** pd-1 inhibitor, hepatocellular carcinoma, liver transplantation

## Abstract

The treatment of recurrent hepatocellular carcinoma (HCC) after liver transplantation is difficult due to the lack of effective treatment options. The available evidence on the emerging immunotherapy in liver transplantation is based on anecdotal experiences and requires additional investigations. To determine the efficacy and safety of immunotherapy in liver transplant recipients, we report three cases of recurrent metastatic HCC after liver transplantation who were treated with nivolumab as off-label salvage therapy.

## Introduction

Hepatocellular carcinoma (HCC) is ranked first among primary hepatic cancers, involving more than half a million patients per year [1]. Several early-phase clinical trials have investigated the value of immunotherapy in advanced HCC, as it represents the example of inflammation-induced cancers [2]. Liver transplantation is the treatment of choice for localized HCC in the absence of contraindications [3]. The recurrence rate after liver transplantation for HCC, despite the strict eligibility criteria, is high, varying between 6% to 18% [4]. The treatment of recurrent HCC after liver transplantation is a real challenge for the clinical practice, given the absence of any therapeutic standards [5].

The principle of immunotherapy consists of the induction of an anti-tumor immune response using monoclonal antibodies targeting immune checkpoints present on the surface of T-cells. Immune-checkpoint inhibitors include two major families of antibodies targeting either the cytotoxic T-lymphocyte-associated antigen 4 (CTLA-4) or the programmed cell death protein 1 (PD-1) [6]. Nivolumab is the first recombinant human monoclonal antibody developed in the world that targets PD-1 [7]. The response rate with this molecule reached 20% with two complete responses in phase I/II trial (CheckMate-040 trial) in advanced HCC. These results seemed extremely promising for the advanced stage of this malignancy known by its poor survival [8]. Nivolumab is also characterized by its persistent effect in responders, which encouraged scientists-clinicians to continue patients’ enrollment for this setting. At the Congress of the American Society of Clinical Oncology (ASCO) in 2017, the updated data of this study confirmed satisfactory results for overall survival (OS) with a median of 28.6 months in the first line of treatment and 15.6 months in the second line [9]. The nivolumab positioning in the first-line versus sorafenib is under investigation in phase III [10].

It is actually known that the tolerance of transplant organs involves the modulation of cell-mediated immunity [11-12] and that the transplant rejection’s risk may be caused by the deregulation of these processes [13]. The fear of transplant rejection leads to the exclusion of the recipient's solid organ transplant from the clinical trials of immune checkpoints inhibitors [14]. The current data on immunotherapy after hepatic transplantation are lacking and are limited to case reports and small published series [15-16].

In this paper, we describe three cases of recurrent metastatic HCC after liver transplantation in whom nivolumab was used as off-label salvage treatment.

## Case presentation

Case reports

Case 1

A 70-year-old male with a personal history of nonalcoholic steatohepatitis presented with multifocal HCC that was diagnosed at the age of 64. No regional lymph node or distal metastasis was found, classified as Child A and model for end-stage liver disease (MELD) 7. He was treated with three courses of arterial chemoembolization, followed by a complete hepatectomy and an orthotopic liver transplant. After 33 months of transplantation, the patient had rising alpha-fetoprotein and the presence of liver metastasis on surveillance imaging. The diagnosis consisted of recurrent metastatic HCC. The patient received sorafenib as first-line treatment. As a second-line treatment, the patient received six cycles of gemcitabine and oxaliplatin. However, his disease progressed according to Response Evaluation Criteria in Solid Tumors (RECIST), and he was treated with regorafenib. After metastatic disease progression, the patient was treated by salvage nivolumab at 240 mg every two weeks. The patient continued the immunosuppression of tacrolimus. The patient presented right-sided abdominal pain, and liver enzymes and total bilirubin were elevated after four doses of nivolumab. The patient was then treated with a pulse of high-dose steroids. Therefore, nivolumab treatment was interrupted. The patient was followed up and died four months after tumor progression.

Case 2

A 62-year-old female with a history of hepatitis B virus and multifocal HCC Child B and MELD 14, underwent liver transplantation. One year after, surveillance imaging revealed adrenal and pulmonary metastasis and mediastinal lymphadenopathy. She received first-line treatment using sorafenib for six months. Because of tumor progression, a second-line treatment by regorafenib was decided. However, the disease progressed and alpha-fetoprotein rose after nine months of second-line tyrosine kinase inhibitor (TKI). She was treated by chemotherapy (5 fluorouracil and oxaliplatin) for six cycles. After progression, a multidisciplinary team-based discussion decided to start nivolumab while maintaining immunosuppression by tacrolimus. She received five cycles of nivolumab 240 mg every two weeks, with continuous monitoring of serum tacrolimus concentration and liver function. No graft rejection or immune adverse events was observed in this case. However, her metastatic disease progressed. A fifth-line treatment by lenvatinib was started. This patient is still alive with clinical-radiological disease stability.

Case 3

This was a 66-year-old male with a history of cirrhosis and hepatitis B virus, with Child B and MELD 20 HCC. After two courses of arterial chemoembolization, a total hepatectomy and orthotopic liver transplant were performed. Two years later, the patient presented with hepatic and pulmonary metastases. The patient was treated with first-line sorafenib for two years. A second-line treatment by Regorafenib was administrated for 9 months. After progression on chemotherapy (gemcitabine and oxaliplatin) used in the third-line, the patient received nivolumab every two weeks. He continued immunosuppression using tacrolimus. The PD-1 inhibition by nivolumab did not induce immune adverse events. His tumor progressed after six cycles of nivolumab and a new therapeutic line using cabozantinib has been started.

## Discussion

Nivolumab was the first PD-1 inhibitor approved for HCC treatment by the Food and Drug Administration (FDA) in September 2017 because of the trial CheckMate 040 outcomes [2-8]. The nivolumab EMA approval has not yet been received even after CheckMate 459 trial data demonstrated clinical improvement in OS and overall response rate (ORR) with nivolumab compared to sorafenib in the first-line setting for advanced HCC [10]. Initially, solid organ transplantations were the exclusion criteria in immune checkpoint inhibitors due to immunosuppressive agents administration [17-18].

The administration of nivolumab after liver transplantation poses the question of its tolerance and efficacity. In our three cases, no severe adverse effects with nivolumab were observed. Notably, liver transplant recipients may, therefore, be included in immunotherapy clinical trials. However, appropriate patient selection, strict laboratory, clinical monitoring, and the use of low immunosuppressive drug dosing during nivolumab therapy are needed.

Immunosuppression and immunotherapy are two crucial conditions in the identification of patients that may receive immunomodulatory agents in this setting [19]. However, the immunogenicity of transplant organs differs. It appears that the liver grafts are less immunogenic as compared to other organs, such as the heart, the kidneys, or the lungs, which leads us to discuss the possibility of less aggressive immunosuppressive treatment use [20].

None of our patients showed a significant benefit under immunotherapy, probably due to a late introduction in the third or fourth-line for aggressive diseases. The small patient sample does not support our conclusion on the efficacy of immunotherapy in hepatic transplant patients. Further studies with a large patient cohort are awaited.

## Conclusions

Overall, the use of immunotherapy in HCC recurrent after liver transplantation is not recommended in light of current data but medicine is one of the most evolving areas where the guidelines change overnight. Therefore, to define the feasibility of immunotherapy in hepatic transplants for HCC, clinical trials including this specific population are needed.
